# Intertidal clams exhibit population synchrony across spatial and temporal scales

**DOI:** 10.1002/lno.11085

**Published:** 2018-12-03

**Authors:** Julie S. Barber, Casey P. Ruff, James T. McArdle, Lindy L. Hunter, Camille A. Speck, Douglas W. Rogers, Courtney M. Greiner

**Affiliations:** ^1^ Fisheries Department Swinomish Indian Tribal Community La Conner Washington; ^2^ Skagit River System Cooperative La Conner Washington; ^3^ Washington Department of Fish and Wildlife Port Townsend Washington

## Abstract

Long‐term datasets can be particularly useful for parsing out factors influencing populations, yet few studies have utilized continuous datasets to quantify population dynamics in bivalve molluscs. We used dynamic factor analysis on a clam biomass dataset spanning 28 yr and five distinct regions in the southern Salish Sea to determine (1) if native intertidal clam populations exhibit synchrony and (2) what environmental covariates may be correlated with these population trends. Once covariates were accounted for, the model with the most data support included three predominant trends to describe multidecadal change in clam biomass. Intraspecific synchrony was highest among Saxidomus gigantea and *Leukoma staminea* populations, with no clear evidence of covariance in Clinocardium nuttallii. Specifically, we quantified a pronounced decadal decline in *L. staminea* and an increase in S. gigantea biomass on most beaches. No beaches showed synchrony in trends across all three species, indicating that species‐specific trends (regardless of location) were more common than beach‐specific trends (regardless of species). Seven environmental covariates were evaluated in their capacity to explain variability in annual mean biomass. Of these, the North Pacific Gyre Oscillation lagged 4 yr prior to the observation year was most supported by the data in the best fitting model, implying that 4 yr old clam biomass is partially determined by oceanographic processes affecting larval clams. Although results suggest large‐scale density‐independent factors play a role in venerid clam population dynamics, it is also likely local factors account for variability not explained by our model.

Research on long‐term population dynamics of commercially and ecologically important species is imperative for the managers, policymakers, and users of these systems (Willis et al. 2007; Giron‐Nava et al. 2017; Hughes et al. 2017). These studies are especially valuable for establishing baselines and developing insight into population patterns and the factors that influence them (e.g., Barry et al. 1995; Menge et al. 2011). In particular, information on local vs. regional or global environmental drivers of long‐term population dynamics can better inform management and conservation efforts (Defeo et al. 2013; Ohlberger et al. 2016; Giron‐Nava et al. 2017). A more comprehensive understanding of the predominant spatial and temporal scales of population synchrony (when populations follow similar patterns), can improve our predictions of how species or ecosystems may respond to anthropogenic, biological, or physical stressors.

Nearshore estuarine ecosystems are affected by a variety of natural factors (e.g., salinity, temperature, competition, larval supply, etc.) and human activity (e.g., fishing effort, shoreline armoring, pollutants, etc.). Due to their highly variable nature, these potential drivers make discerning trends in estuarine communities especially difficult (Dethier and Schoch 2005). Despite these challenges, quantifying population level change in estuarine communities is worthwhile because of the ecosystem services and functions provided by species in these coastal environments.

Bivalves are a particularly significant group of estuarine species because of their ecological, cultural, and economic importance. Among other factors, these molluscs are substantial consumers of phytoplankton (providing essential filtration and biodeposition services); considerable economic drivers of nearshore fisheries; cultural keystone species integral to the health and wellbeing of Indigenous communities; and valuable food sources for predators such as worms, fishes, and birds (Garibaldi and Turner 2004; Newell 2004). Long‐term studies have illustrated the effects of some biological and physical variables on the population dynamics of estuarine bivalves. For example, decadal studies in the Netherlands have demonstrated important connections between recruitment and temperature with clam species that are valuable to commercial fishers as well as to shellfish‐consuming birds (Philippart et al. 2003; Beukema et al. 2010). Upwelling, river discharge, and the North Atlantic Oscillation affect Portuguese lagoon and coastal bivalve populations differently (Baptista and Leitão 2014; Baptista et al. 2014). Oyster population dynamics in New Jersey, U.S.A. have shown regime shifts in response to disease; and invasive bivalve species have been able to displace or outcompete native bivalve species, generating functional changes within particular ecosystems (Pranovi et al. 2006; Powell et al. 2008; Novoa et al. 2016). While these studies and others have contributed to a growing understanding of factors influencing bivalve population dynamics, few utilize continuous datasets spanning decades to describe change on relatively large spatial scales (but *see* Baptista and Leitão 2014).

Improving our knowledge of population synchrony in understudied bivalve species is especially important in regions where these species support valuable fisheries and fill crucial niches in ecological communities. For example, if managers can better understand local or regional drivers of populations, they may be able to predict when certain environmental changes will negatively impact populations and adjust management regimes accordingly (e.g., Ward et al. 2010). Despite the advantages of understanding population drivers of bivalves, we do not know how multi‐regional environmental conditions structure population synchrony (or lack thereof) in a system, although we do know that physical gradients play a role in determining the biota found in estuaries (e.g., Dethier and Schoch 2005; Dethier 2010).

The Salish Sea, a network of waterways between Washington, U.S.A. and British Columbia, Canada, contains large populations of native and non‐native venerid clam species. These species make up a significant portion of the biomass on pebble‐sand beaches and are the basis for popular recreational and commercial intertidal fisheries in Washington (Dethier 2006). Four native species (*Leukoma staminea*, *Saxidomus gigantea*, *Clinocardium nuttallii*, and *Tresus capax*) and one non‐native species (*Ruditapes philippinarum*) compose much of the fisheries‐targeted intertidal clam biomass. While there is evidence from discrete data collections that *L. staminea* populations are declining throughout the range of the species (Dunham et al. 2007; Shigenaka et al. 2008; Novoa et al. 2016; Strickland et al. 2016), very little quantitative information exists on continuous long‐term population trends of these clam species. Because the southern Salish Sea can be divided into different sub‐basins, which are largely defined by oceanographic processes, the region can serve as a natural laboratory within which to analyze spatial and temporal trends in bivalve population synchrony. Furthermore, few studies have attempted to quantify what environmental drivers (if any) influence the long‐term trends seen in these populations.

Multivariate time‐series analyses are one way to describe decadal change in populations while incorporating covariates to detect potential relationships between environmental factors and populations. Dynamic factor analysis (DFA) is a dimension‐reduction technique that can be applied to ecological time‐series data, such as animal biomass, to identify common population trends and the degree to which individual populations are associated with a specific trend (Zuur et al. 2003). Importantly, DFA can be used to evaluate the relative degree of covariance between a suite of ecological time series (e.g., Ohlberger et al. 2016; Freshwater et al. 2017; Ruff et al. 2017). While DFA has been used to study the effect of environmental parameters on commercial catch rates of clams in Portugal (Baptista and Leitão 2014; Baptista et al. 2014), to our knowledge, DFA has never been applied to clam population data to better understand population synchrony. Thus, DFA provides us with the unique opportunity to quantify temporal and spatial relationships in southern Salish Sea clam populations and improve our ability to manage populations of these important species.

The primary goal of our research was to quantify population trends in Salish Sea native venerid clam species. We used DFA to address the following questions: (1) do native intertidal clam populations exhibit synchrony across spatial and temporal scales and (2) do any large‐scale environmental factors influence these population trends? Specifically, we assessed whether population trends in native clams grouped by biological factors (e.g., inter‐ or intraspecifically) and/or physical factors (e.g., southern Salish Sea sub‐basins, individual beaches), and if the addition of various biological or physical covariates better described variability in clam biomass across space and through time.

## *Material and methods*

### Site description

**Figure 1:**
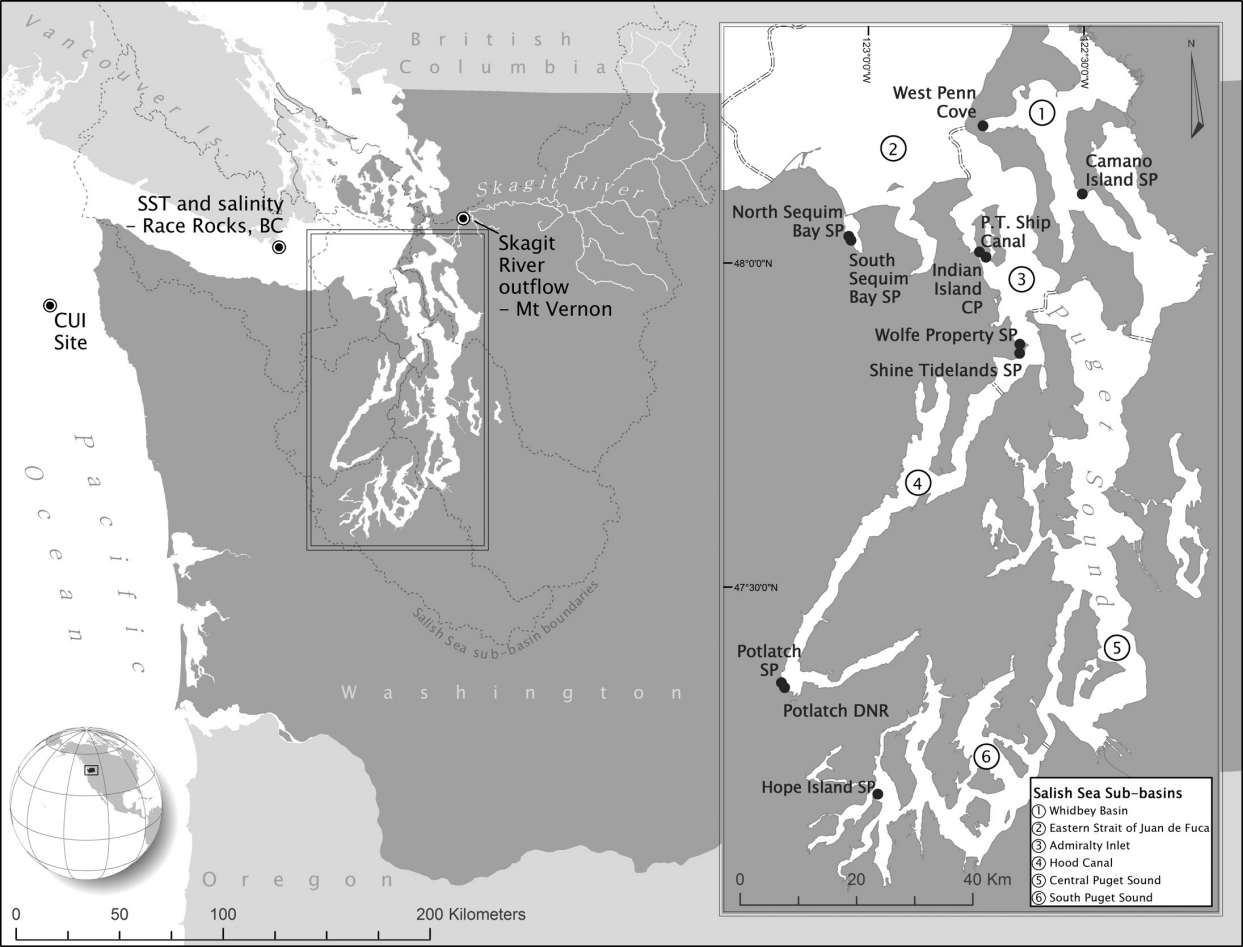
The southern Salish Sea with locations of survey beaches and covariate monitoring stations. SST, sea surface temperature; CUI, coastal upwelling index used for determining spring transition date; DNR, Department of Natural Resources land; SP, state park; CP, county park. Dashed double line indicates sub‐basin boundaries.

The southern Salish Sea contains the marine inland waters of Washington, including Puget Sound and the Strait of Juan de Fuca (Fig. 1). Puget Sound is a partially mixed estuarine fjord system that can be separated into sub‐basins primarily defined by oceanographic processes (Khangaonkar et al. 2011) (Fig. 1). Typical of estuarine fjords, these sub‐basins are divided by a series of sills which impact the flow pattern into and out of the interconnected basins, with the majority of water entering Puget Sound from the Strait of Juan de Fuca over a double sill in Admiralty Inlet. Khangaonkar et al. (2011) demonstrated that circulation patterns in Puget Sound can vary from well‐mixed, fast‐moving flow (e.g., Admiralty Inlet) to more stratified flow in slower‐moving sub‐basins (e.g., Hood Canal, Whidbey Basin); this stratification and mixing is created by tidally averaged circulation and flushing (two daily unequal tides), not tidal currents. Southern Hood Canal is known for its high water residence time while Whidbey Basin has moderate retention times and Admiralty Inlet has a relatively short residence time (max annual deep water residence time = 99, 41, and 23 d, respectively; Babson et al. 2006). Salinity and water temperature patterns in Puget Sound are largely correlated with local freshwater inflow and air temperatures, respectively, and oceanographic properties are more influenced by local environmental parameters than large‐scale climate variations (Moore et al. 2008). One important regional parameter is freshwater input, which is heavily influenced by Skagit River discharge (located in Whidbey Basin, Fig. 1) with peaks in the winter and late spring/early summer (Babson et al. 2006).

### Clam surveys

Following similar sampling methods, the Washington Department of Fish and Wildlife (WDFW) has conducted surveys on the same beaches since 1977 to determine clam biomass for management purposes (Campbell 1996). All of these beaches have dense clam populations capable of supporting recreational clam harvest and, while substrate type varies by beach, the locations share substrates preferred by venerid clams in the Salish Sea (Kozloff 1983). For this analysis, we selected 11 beaches with relatively consistent data collection from 1989 to 2016, located within five different southern Salish Sea sub‐basins: Whidbey Basin, eastern Strait of Juan de Fuca, Admiralty Inlet, Hood Canal, and South Sound (Table 1; Fig. 1). These beaches had a minimum of 14 yr of discrete sampling dates (mean = 23.5 yr) within the 28‐yr study period (Table 1). One of our study sites, Camano Island State Park (CA) has been closed to recreational clam harvest since 2002, although ceremonial and subsistence tribal treaty harvest still occurs at this location. Despite the proximity of several beaches to one another (e.g., WP and ST), nearby beaches did not always share physical attributes such as fetch, beach slope, alluvial processes, freshwater and estuarine input, or wave dynamics.

**Table 1:** Beach survey information including the number of survey years utilized in dynamic factor analysis, the mean survey tide height (relative to mean lower low water) and area, and the mean biomass (g/m^3^) for each of the three study species averaged across all years.

						Mean biomass (g/m^3^) ± SE		
Beach name	Abbreviation	Sub‐basin	# of survey years	Mean survey tide height (m) ± SE	Mean survey area (ha) ± SE	*S. gigantea*	*L. staminea*	*C. nuttallii*
Camano Island State Park	CA	Whidbey	20	−0.82±0.14	5.70±0.27	2389.61±355.60	529.57±112.89	232.18±32.31
West Penn Cove	WC	Whidbey	16	−0.78±0.19	10.75±0.65	1343.38±222.43	716.58±117.89	157.28±23.03
Sequim Bay State Park (South)	SS	Strait of Juan de Fuca	26	−0.51±0.11	1.51±0.12	302.14±56.78	1264.23±118.60	155.40±29.62
Sequim Bay State Park (North)	SN	Strait of Juan de Fuca	24	−0.29±0.58	1.89±0.12	461.12±96.77	1343.52±84.11	127.62±19.22
Indian Island County Park	I I	Admiralty	25	−0.69±0.12	9.86±0.38	321.64±41.49	223.95±20.07	36.49±3.85
Port Townsend Ship Canal	PT	Admiralty	27	−0.67±0.25	4.22±0.46	789.61±127.28	145.72±11.13	52.36±9.23
Wolfe Property State Park	WP	Admiralty	26	−0.51±0.12	4.24±0.27	327.21±26.61	242.09±20.95	13.55±2.90
Shine Tidelands State Park	ST	Admiralty	28	−0.68±0.18	3.93±0.1	1013.52±111.83	483.03±45.84	56.40±8.13
Potlatch State Park	PS	Hood Canal	27	−0.88±0.14	13.36±0.63	134.96±21.25	103.67±9.59	39.95±5.65
Potlatch Dept. Natural Resources	PD	Hood Canal	25	−0.73±0.11	5.35±0.28	487.33±63.92	159.89±17.28	108.98±20.45
Hope Island Marine State Park	HI	South Sound	14	−0.84±0.28	5.77±0.32	450.74±39.92	119.71±16.49	9.06±3.48

SE, standard error.

Annual beach survey methods (detailed in Campbell [1996] but updated here and in Barber et al. [2012]) delineated an area using property boundaries, the upper clam habitat boundary, and the waterline (e.g., −0.6 m relative to mean lower low water [MLLW]) or lower clam habitat boundary. For each beach, surveys were conducted no more than 2 h before and after the lowest low tide of the day and targeted the same tide height (relative to MLLW) each year (Table 1). Within the delineated area, various grids were designed for individual beaches using evenly spaced transects (e.g., 30.5 m apart) set perpendicular to the shoreline starting from a random point, with quadrats also starting from a random point but then spaced evenly (e.g., 12.2 m) along each transect line (e.g., grid size = 30.5 × 12.2 m). Specific sites marked along transect lines identified quadrat locations where a 0.093 m^3^ area was dug. Clams within these quadrats were collected, identified, measured, and weighed whole‐live or whole‐frozen (Bradbury et al. [2005] found a negligible, < 1%, difference between whole‐live and whole‐frozen clam weight). Although the surveys targeted legal‐sized adult clams (> 38 mm), juvenile or smaller adult clams were collected if observed. In 2002, WDFW began a sub‐sampling process on the beaches where clams from only one out of every two or three quadrats were collected for length‐weight measurements. When shell valves were broken and/or specimen weight was missing, beach or region‐specific length‐weight models were used to calculate individual clam weight (Bradbury et al. 2005; Barber et al. 2012). If a clam was too broken to weigh or measure, we assigned that individual the mean weight of its species from the survey that year.

Some of the beaches in this analysis contained physical features (e.g., stream mouth, extremely soft silt, etc.) that prevented surveying the entire beach from property boundary to boundary; these areas were consistently skipped during the surveys. Additionally, over the 28‐yr span, WDFW had to alter certain aspects of the survey design on some beaches including minor loss of survey area due to changes in property boundaries or natural changes in coastal geomorphology (e.g., natural estuarine expansion, etc.); only beaches with minor changes or no changes were included in this analysis.

We selected three native clam species for this study (butter clams = *S. gigantea*, native littlenecks = *L. staminea*, and heart cockles = *C. nuttallii*) and calculated mean biomass (g/m^3^) for each species and year a beach was surveyed. The non‐native Manila clam, *R. philippinarum*, was not included in the analysis because this recreationally important species either was not found on the study beaches or populations were enhanced on beaches several years past the start of this analysis.

### Covariates

The addition of covariates to this study was largely exploratory due to the lack of information on how environmental factors may influence population dynamics of *S. gigantea*, *L. staminea*, and *C. nuttallii*. Furthermore, covariates could influence clam life history phases disparately, where some explanatory variables are likely to influence larval survival and others could affect adult populations. As a result, we tested 1–5 yr lags for each covariate applied to the model to determine appropriate lag structures for these three species. This lag structure was selected to encompass the age of first reproduction through to 4–5 yr old clams which are generally larger in size and less susceptible to external stressors. *S. gigantea*, *L. staminea*, and *C. nuttallii* reach sexual maturity by 1, 2–3, and 2 yr, respectively (Gallucci and Gallucci 1982; Cheney and Mumford 1986; Chew and Ma 1987). We did not fit models using annual lags of zero for the environmental covariates due to the presumed negligible effect on already settled adult clams (i.e., > 5 yr old).

We selected the following seven covariates for use in the model based upon (1) known effects of these covariates on different bivalve species located in other geographic regions or (2) published literature demonstrating how environmental variables influence physical and biological factors of the Salish Sea: sea surface temperature (SST) and salinity (SSS), Skagit River flow, air temperature, the North Pacific Gyre Oscillation (NPGO), El Niño (ENSO), and spring transition from downwelling to upwelling (Table 2). Our analysis only considered the singular effects of covariates rather than their combined effects.

**Table 2:** Information on environmental covariates used in dynamic factor analysis including how data were summarized and used in the model (e.g., mean winter, spring, and summer SST).

Covariate	Data summary	Station location	Source
Sea surface temperature (SST)	Seasonal means: Winter (win) = Jan–Mar, Spring (spr) = Apr–Jun, Summer (sum) = Jul–Aug	Race Rocks, Strait of Juan de Fuca	http://www.pac.dfo‐mpo.gc.ca/science/oceans/data‐donnees/lightstations‐phares/data/RaceRocksDailySalTemp.txt
Sea surface salinity (SSS)	Annual mean	Race Rocks, Strait of Juan de Fuca	http://www.pac.dfo‐mpo.gc.ca/science/oceans/data‐donnees/lightstations‐phares/data/RaceRocksDailySalTemp.txt
Skagit River outflow (flow)	Seasonal means: Spr = Mar–Jun, Sum = Jul–Sep	Site # 12200500 Mt. Vernon, Washington	http://waterdata.usgs.gov/nwis/dv
Air temperature (AirTemp)	Annual mean	Puget Sound lowlands	https://www.ncdc.noaa.gov/cag/time‐series/
North Pacific Gyre Oscillation (NPGO)	Annual mean from Jul to Jun	NA	http://www.oces.us/npgo/npgo.php
El Niño (ENSO)	Annual mean from Jul to Jun	NA	http://www.esrl.noaa.gov/psd/enso/mei/table.html
Spring transition index (STI)	Date of spring transition from downwelling to upwelling	Fig. 1, off northwestern Washington, “CUI Site”	https://www.pfeg.noaa.gov/products/PFELData/upwell/daily/p06dayac.all

Sea surface temperature was selected because several studies on other clam species have shown that SST can affect clam growth, recruitment, and mortality (e.g., Philippart et al. 2003; Beukema et al. 2009; Narváez et al. 2015). Sea surface salinity is known to influence the availability of high‐quality particulate organic matter (Lowe et al. 2016), and SSS and river discharge can affect clam growth and population structure (e.g., Defeo and de Alava 1995; Marsden 2004; Baptista et al. 2014). We included air temperature as a covariate because intertidal bivalves are exposed to atmospheric conditions twice a day during low tides (Dethier and Schoch 2005). Although the SST and SSS monitoring station is located in the Strait of Juan de Fuca (Fig. 1), we believe the variability in these data still serves as a proxy of the variability in SST and SSS experienced by more than half of our study beaches. Puget Sound lowland air temperature and Skagit River flow were also included in the model because variability in Puget Sound SST and SSS is best explained by anomalies in these two respective explanatory variables (Moore et al. 2008). Several studies have linked the NPGO or ENSO with invertebrate recruitment or productivity (Urban and Tarazona 1996; Menge et al. 2011; Sydeman et al. 2013) and, importantly, the NPGO is a good proxy for nutrient and chlorophyll levels which affect bivalve food sources (Di Lorenzo et al. 2008). Shanks and Roegner (2007) found that commercial Dungeness crab, *Metacarcinus magister*, catch in Washington State was negatively correlated with the date of the spring transition from downwelling to upwelling. Because Dungeness crab predation can affect post‐settlement mortality of clams and since oceanic Dungeness crab are found within Puget Sound (Boulding and Hay 1984; Dinnel et al. 1993), upwelling could indirectly affect clam biomass in the southern Salish Sea. While other covariates such as chlorophyll *a* (Chl *a*), pH, fishing effort, or bioturbator biomass would have been extremely valuable in this analysis, to the best of our knowledge, no continuous record of these covariates in the southern Salish Sea exists from 1989 to 2016. Because DFA cannot work with missing data in the covariate time series, we were limited to selecting environmental covariates that had continuous datasets spanning 28 yr.

### Modeling approach

In DFA, the time series of observed data are treated as a linear combination of one or more unobservable trends, which are modeled separately as one or more random walk processes. This framework is a form of a state‐space model because it estimates both observation variance and process variance separately. Observation variance is commonly influenced by measurement error due to non‐exhaustive sampling designs, as in the case of this study, whereas process variance is influenced by environmental stochasticity. DFA has been shown to be useful in its application to time series of ecological data in evaluating both hypothesis‐driven and exploratory analyses of population covariance (Zuur et al. 2003; Jorgensen et al. 2016; Freshwater et al. 2017).

The observed time series (**y**) are modeled as a linear combination of up to *m* shared hidden trends (**x),** explanatory variables (**d),** and observation errors (**v):**

(1) $$y_{t}\;\;=\;\;{\mathrm{Zx}}_{t}+{\mathrm{Dd}}_{t}+v_{t}$$

where **y**_t_ is an *n* × 1 vector of the observed natural log of clam biomass for each of *n* = 33 (three species × 11 sites) discrete surveyed populations at time *t*, **Z** is an *n* × *m* matrix of factor loadings that indicate how much temporal variability in each time series is explained by each of *m* unobserved trends modeled, and **x**_t_ is an *m* × 1 vector of hidden states at time *t*. For this analysis, we considered two forms of **Z**, one in which each population is assigned a unique factor loading on each of the unobserved trends being modeled, and the other in which populations of a given species occurring at beaches in close geographic proximity (i.e., SS‐SN, II‐PT,WP‐ST, PS‐PD; Fig. 1) are assigned equal factor loadings on each trend. Under the first form, we assume that each population exhibits its own unique pattern in biomass over time, regardless of location. For the latter form of **Z**, we assume that clam populations of a given species at each pair of neighboring beaches may be subject to similar demographic rates resulting in these populations exhibiting similar patterns in biomass over time. Therefore, in this latter form, **Z** is constrained such that respective rows representing populations of a species occurring at a pair of neighboring beaches are assigned identical values. Ultimately, models including this latter form of **Z** fit the observed data better (Supporting Information Table S1); subsequent discussion will focus on this form.

**Z** is constrained further such that in the first *m* – 1 rows of the matrix, the factor loadings in column *j* and row *i* are set to zero where *j* > *i*. This constraint is necessary for model identifiability and does not affect the interpretation of model results due to the post‐fitting varimax rotation of the factor loadings. The observation model assumes that the time series have a mean of zero; therefore, the observed data are scaled to a mean of zero and standard deviation of one. Because we allowed only one covariate to be included in the model at a time, individual covariates enter the model as ***d***_*t*_, a 1 × 1 scalar of the covariate value in year *t*, and **D** is an *n × 1* vector of the estimated effect size of the covariate on each population. Most of the environmental covariates evaluated can influence environmental conditions over broad spatial scales and may influence clam population dynamics similarly regardless of geographic location. Therefore, **D** is constrained to include only three unique parameters to represent the effect sizes on each of the three species independent of location. Observation errors, ***v***_*t*_, are assumed to be distributed as a multivariate normal with *n* × 1 mean vector 0 and an *n* × *n* variance–covariance matrix **R.** Because this study includes three different species of clams that occupy different locations within the intertidal zone, we constrained **R** such that the variance of the observation errors (the diagonal elements of **R**) differed between each species with no covariance among populations. The unobserved trends (**x**) are modeled as *m* random walk processes:

(2) $$x_{t}\;\;=\;\;x_{t-1}+w_{t}$$

where process errors, **w**_t_, are assumed to be distributed as a multivariate normal with *m* × 1 mean vector 0 and an *m* × *m* identity matrix **I** with a variance of one and no covariance between each hidden trend. The vector of *m* initial states at year *t* = 0 (**x**_0_) is distributed as a multivariate normal with a mean of zero and an *m* × *m* variance–covariance matrix with large variances (the diagonal elements are set equal to 10) and no covariance between each hidden trend. The population‐specific model fits are the product of the factor loadings and the trends in clam biomass (**Z** × **x**) plus the effects of any environmental covariates. Model parameters were estimated in a maximum likelihood framework using the MARSS package developed for the R programming environment (Holmes et al. 2012, 2018; R Core Team 2017).

We used DFA to identify up to three common trends among 33 time series of mean clam biomass distributed across the study area spanning years 1989–2016 (Table 1; Fig. 1). After fitting the trend‐only DFA models, we fit models allowing up to one covariate at a time in each model in addition to the trends. We evaluated the data support for each model using the Akaike information criterion corrected for small sample size (AICc; Burnham and Anderson 2002). The model with the lowest AICc value was identified as the model with the strongest data support although models with a ΔAICc less than two were considered to have similar support. For each model, after confirming that convergence was achieved for all parameters, model residuals were examined to confirm whether assumptions of normality and independent observation errors were met and that residuals were not autocorrelated (Supporting Information Figs. S1–S4). Variances for the maximum likelihood estimates of model parameters were calculated using the Hessian approximation (Harvey 1989).

A repository with detailed code and raw data can be accessed at https://doi.org/10.6084/m9.figshare.6834935.v1.

### Evaluating covariation in clam biomass

We relied on three complementary approaches to evaluate covariation between clam populations with respect to their variability in biomass. First, we quantified the correlation (Spearman's rank coefficient) between each observed time series to determine whether there were any predominant patterns of synchrony (e.g., species or regional). Second, for DFA models with more than one trend, we applied a varimax rotation to the factor loadings to maximize the variance along one factor/trend. Factor loadings were rotated by multiplying the inverse *m* × *m* rotation matrix (**H** ^−1^ **)** by the factor loadings (**H** ^**−1**^ × **Z)** and the trends were rotated by multiplying the rotation matrix by the trends (**H** × **x)**. The varimax rotation assigns a higher factor loading (positive or negative) to those trends that explained a larger portion of the temporal variability for a specific time series. In cases where a specific population received a strong negative loading on a trend, the trajectory of the population was the inverse of the trend. These steps enabled the matrix of population‐specific factor loadings to be more easily related to each of the trends. Finally, following Jorgensen et al. (2016), we quantified the correlation (Spearman's rank coefficient) between each observed time series of clam biomass after accounting for environmental covariate effects and each of the trends. For each of the populations, the correlations among the trends reflected the degree of association between a population and a trend such that higher correlations reflected stronger associations between a population and a trend, whereas low correlations indicated little or no association with a trend. We focused on whether each of the three species were more associated with a given trend by comparing the resulting distribution of correlation coefficients for each species across each of the estimated trends.

## *Results*

**Figure 2:**
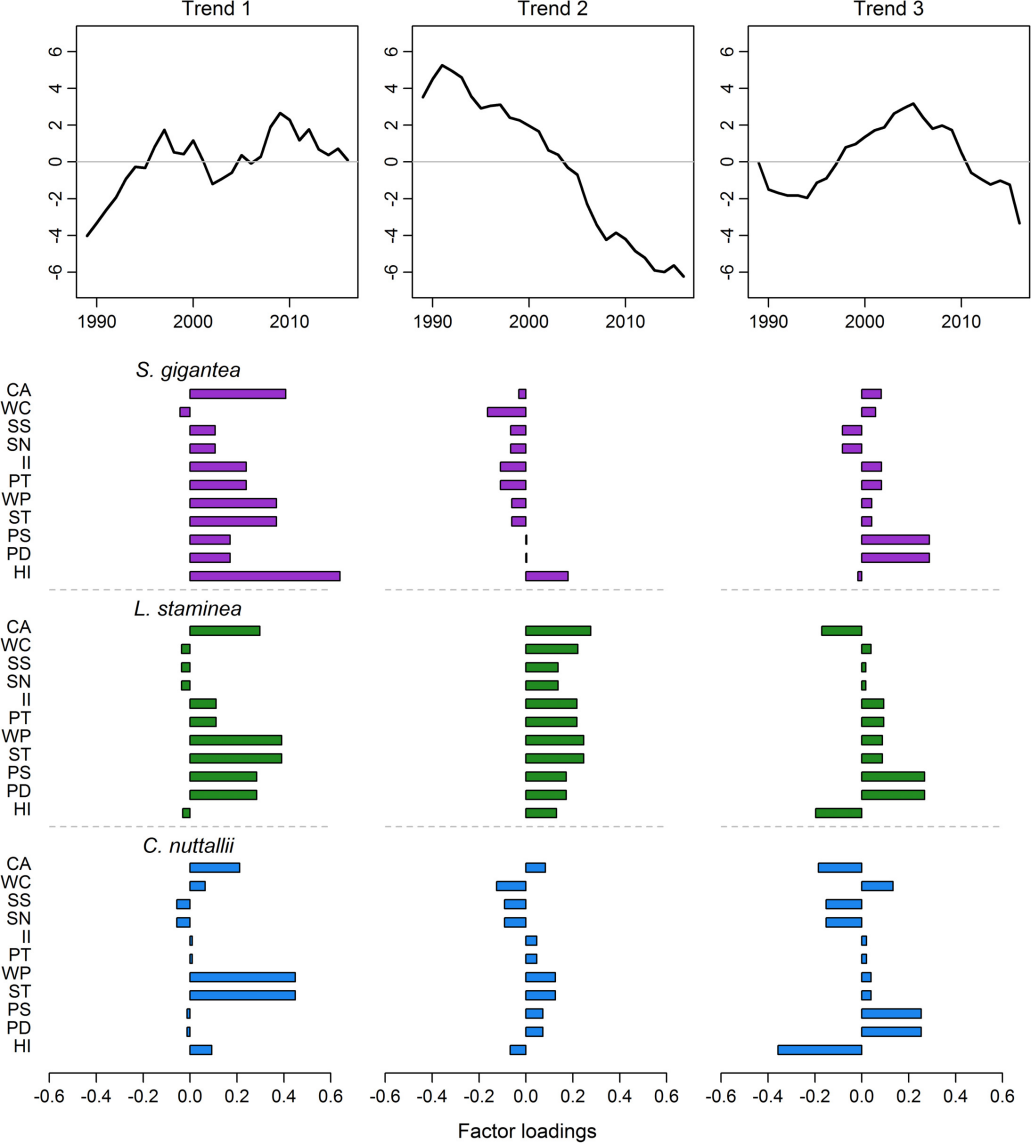
Estimated trends (top row) and factor loadings per trend (bottom row) for the best‐fitting model (three trends and the NPGO lagged 4 yr) (Table 3). Negative factor loadings indicate that the trend is inverted. Bars for the factor loadings are color coded by species, S. gigantea = purple, *L. staminea* = green, and C. nuttallii = blue. See Table 1 for a key to beach name abbreviations.

The DFA model most supported by the data included three common trends in biomass among the 33 clam populations plus the effects of the NPGO lagged 4 yr (Table 3; Fig. 2). Of the seven environmental covariates evaluated, the NPGO added the most explanatory power, showing strong positive associations with the biomass of each species. Generally, trend 1 was characterized by a cyclical temporal pattern in clam biomass with peaks in biomass occurring in years 1997 and 2009, followed by a decline through 2016. Trend 2 was characterized by an overall decline in clam biomass throughout the duration of the time period from 1991 through 2016. Trend 3 was characterized by an overall increase in biomass from 1989 until 2005 followed by a decline to below average levels through year 2016 (Fig. 2).

**Table 3:** Top 15 DFA models ranked in order of decreasing data support (increasing AICc). Models were fit assuming shared observation variance among species but no covariance. Models without covariates were included in the analysis but received less support from the data. Covariate abbreviations are defined in Table 2.

Model	k	Delta AICc	AICc weight	Median covariate effect size (95% CI)		
				*S. gigantea*	*L. staminea*	*C. nuttallii*
3 trends + NPGO.lag.4	66	0.00	7.43E‐01	0.38 (0.26,0.51)	0.15 (0.01,0.29)	0.19 (0.07,0.31)
2 trends + NPGO.lag.4	47	3.18	1.51E‐01	0.24 (0.13,0.35)	0.1 (−0.03,0.23)	0.11 (0,0.23)
2 trends + airTemp.lag.3	47	7.01	2.23E‐02	−0.09 (−0.19,0.01)	−0.12 (−0.24,‐0.01)	−0.16 (−0.27,‐0.04)
2 trends + NPGO.lag.5	47	7.56	1.70E‐02	0.24 (0.12,0.35)	−0.02 (−0.15,0.11)	0.03 (−0.09,0.15)
2 trends + STI.lag.1	47	8.91	8.62E‐03	−0.07 (−0.17,0.03)	−0.17 (−0.28,‐0.06)	0 (−0.12,0.11)
2 trends + STI.lag.2	47	8.94	8.49E‐03	−0.11 (−0.21,‐0.02)	−0.1 (−0.21,0.01)	−0.12 (−0.24,‐0.01)
2 trends + airTemp.lag.5	47	9.07	7.96E‐03	0.07 (−0.03,0.17)	0.14 (0.03,0.25)	0.13 (0.02,0.25)
3 trends + NPGO.lag.5	66	9.87	5.34E‐03	0.24 (0.13,0.35)	−0.02 (−0.14,0.11)	0.03 (−0.08,0.15)
2 trends + flow_spr.lag.1	47	10.16	4.63E‐03	−0.09 (−0.18,0.01)	−0.15 (−0.25,‐0.04)	0.04 (−0.07,0.16)
3 trends + airTemp.lag.3	66	10.63	3.66E‐03	−0.1 (−0.19,0)	−0.13 (−0.24,‐0.02)	−0.15 (−0.27,‐0.04)
3 trends + STI.lag.2	66	10.63	3.64E‐03	−0.13 (−0.22,‐0.04)	−0.11 (−0.22,‐0.01)	−0.13 (−0.24,‐0.02)
2 trends + NPGO.lag.1	47	11.30	2.61E‐03	−0.19 (−0.32,‐0.06)	−0.09 (−0.22,0.05)	−0.01 (−0.14,0.11)
2 trends + sal.lag.3	47	11.93	1.91E‐03	0.16 (0.04,0.28)	0.08 (−0.04,0.2)	0.02 (−0.1,0.14)
3 trends + NPGO.lag.3	66	11.95	1.89E‐03	0.12 (0,0.23)	0.17 (0.03,0.3)	0.13 (0.01,0.25)
3 trends + airTemp.lag.5	66	12.33	1.56E‐03	0.13 (0.02,0.23)	0.19 (0.09,0.3)	0.16 (0.05,0.27)

lag.#, number of years lagged.

**Figure 3:**
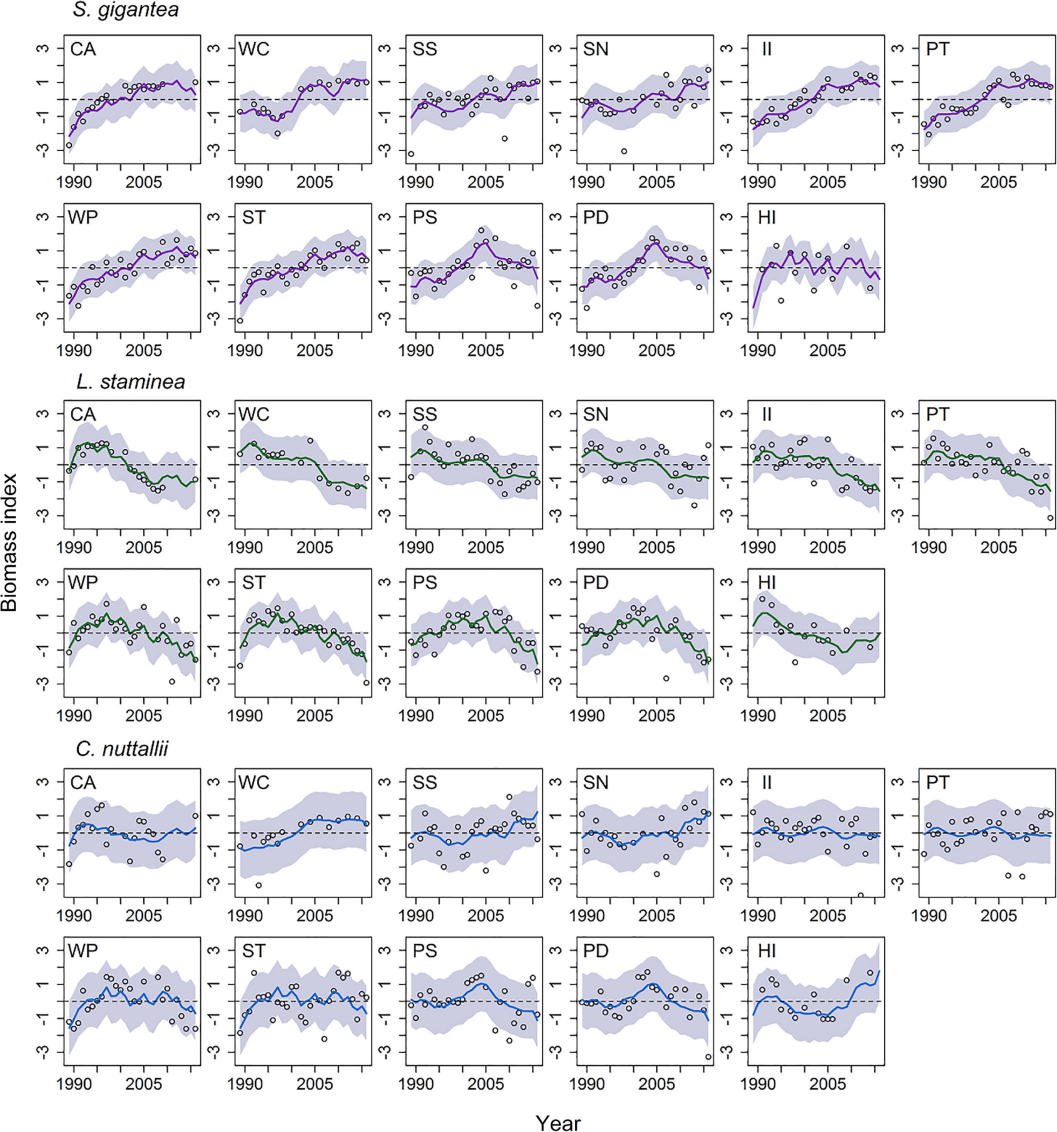
Dynamic factor analysis results for the best‐fitting model: model fitted values (lines) and observed clam biomass (open circles) for S. gigantea (purple lines), *L. staminea* (green lines), and C. nuttallii (blue lines). Shading indicates the 95% confidence intervals of the model fitted values. See Table 1 for a key to beach name abbreviations.

**Figure 4:**
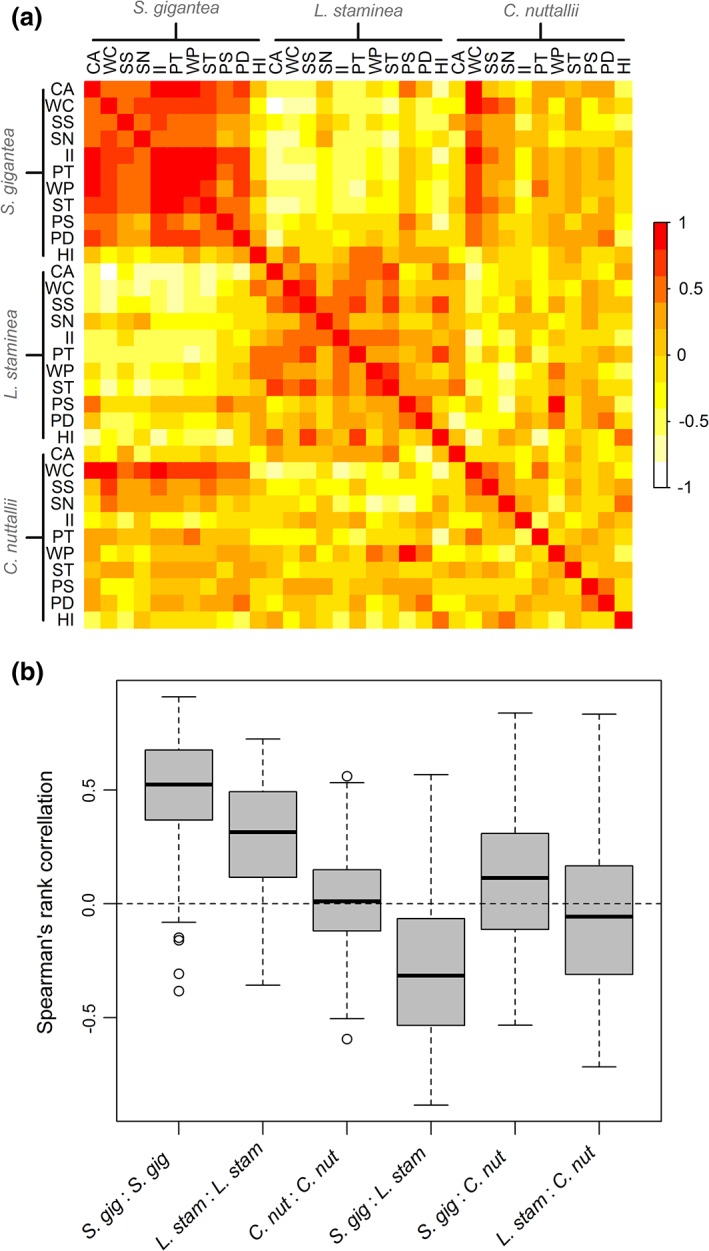
(**a**) Pairwise Spearman's rank correlations among time series of observed clam biomass between 1989 and 2016 for S. gigantea, *L. staminea*, and C. nuttallii. The color of each box shows the strength of the correlation between a population in row *i* and a population in column *j* of the matrix. See Table 1 for a key to beach name abbreviations. (**b**) Boxplots summarizing the distribution of correlation coefficients among time series of observed clam biomass. Each rectangle spans the interquartile range (IQR) of the correlation coefficients and the median is shown by a thick line. Whiskers include data within 1.5 × IQR of each quartile beyond which outliers are shown as open circles.

For *S. gigantea* and *L. staminea*, we found strong evidence for intraspecific population synchrony in biomass trends regardless of geographic location within the southern Salish Sea (Figs. 3, 4). Except for *C. nuttallii*, correlations between populations within a species were less variable than correlations between species (Fig. 4). Specifically, correlations among populations of *S. gigantea* were stronger (median correlation = 0.52), followed by *L. staminea* (median correlation = 0.31), and the weakest for *C. nuttallii* (median correlation = 0.01) (Fig. 4). In terms of DFA factor loadings, *S. gigantea* either exhibited strong positive loadings on trend 1 and/or some combination of positive or negative loadings on trends 2 and 3; this translated to an overall increasing trend on eight out of the 11 populations, a decreasing trend in the last decade for PS and PD, and an increase to relatively stable population for HI (Figs. 2, 3). Since approximately 2012–2013, *S. gigantea* biomass appears to level off and then decline on several beaches (CA, II, PT, WP, ST). In contrast, the majority of *L. staminea* populations exhibited either strong positive loadings on trend 2 or a combination of positive loadings on trend 2 and negative or positive loadings on trends 1 or 3. This translated to an overall declining trend for the majority of beaches or a declining trend since 2005 for PS and PD (Figs. 2, 3). Of the three species, *C. nuttallii* exhibited the largest variation with respect to its associations with each of the three trends across the 11 populations (Figs. 2, 3). Moreover, the only incidence of beach‐specific synchrony appears to occur between the two Hood Canal sites (PS and PD), where all three species exhibited an overall increasing trend in biomass from 1989 through 2005 followed by a decline through 2016 (Fig. 3).

**Figure 5:**
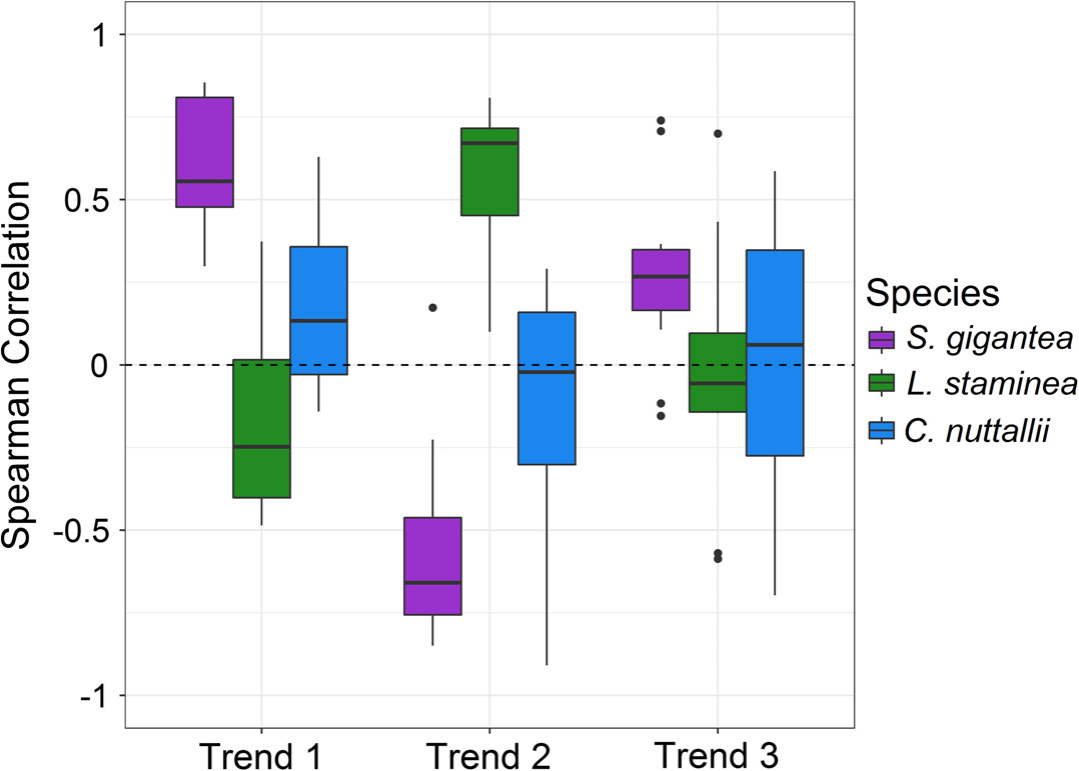
Spearman's rank correlations between the observed clam biomass and the three trends produced from the best‐fitting DFA model (Model 1, Table 3) for S. gigantea, *L. staminea*, and C. nuttallii. Boxplots summarize the distribution of correlation coefficients between each species and trend. Each rectangle spans the interquartile range (IQR) of the correlation coefficients and the median is shown by a thick line. Whiskers include data within 1.5 × IQR of each quartile beyond which outliers are shown as filled circles.

Correlations between the observed time series and each of the three modeled trends showed similar species‐specific patterns to the factor loading patterns (Fig. 5). *S. gigantea* populations had the strongest positive correlations with trend 1 (median correlation = 0.55) indicating that populations were generally associated with an increasing trend, whereas *L. staminea* had the strongest positive correlations with trend 2 (median correlation = 0.67) indicating that populations were generally associated with a declining trend. In contrast to the other two species, *C. nuttallii* populations did not exhibit a clear pattern of association with any of the three trends although populations tended to be more correlated with trend 1 than the other two trends (median correlation = 0.13).

## *Discussion*

Our results indicate that species‐specific population synchrony is more common in the southern Salish Sea than beach or sub‐basin specific patterns. The relatively strong intraspecific synchrony in *S. gigantea* and *L. staminea* populations imply that large‐scale factors are likely driving components of the common trends seen in these species. The Moran effect, where spatially distinct populations exhibit synchrony due to large‐scale density‐independent factors (e.g., climate), is one likely explanation for these population trends (reviewed in Bjørnstad et al. 1999; Liebhold et al. 2004). The patterns for *C. nuttallii* generally show lower synchrony, indicating that local factors, rather than a phenomenon such as the Moran effect, could be influencing population dynamics for this species. It is also possible, however, that the lower overall abundance of *C. nuttallii* on the beaches (Table 1) lead to greater variability in the data, making it harder to detect a synchronous pattern in biomass for this species.

The pronounced multidecadal decline in *L. staminea* is of particular concern as similar declines of this species have been reported from northern Baja California to the Aleutian Islands (Dunham et al. 2007; Shigenaka et al. 2008; Novoa et al. 2016; Strickland et al. 2016). These studies, however, relied on data from discrete sampling events with many years (sometimes decades) missing between samples. Conversely, our study used relatively continuous data to document a pronounced biomass decline following one of two similar patterns (gradual decline since 1991 or slight increase to 2005 and then decline, Fig. 3). To our knowledge this is the first study to combine evidence throughout the range of this species and, importantly, conclude that *L. staminea* populations are likely declining across their entire range. This result corroborates conclusions from the previous studies but it likely alters how these studies may have interpreted reasons for the decline. For example, Novoa et al. (2016) suggests habitat loss, lowered salinity, and an increase in non‐native species as potential reasons for the decline of *L. staminea* in southern California, while Dunham et al. (2007) questioned whether overharvesting or other human activities could explain the decline in British Columbia. Suggestions such as these were based on more local factors, whereas our research implies that one or more large‐scale factor(s), such as disease, oceanographic processes, or climate change, are driving the concerning pattern of extensive biomass loss seen in this species.

While the aforementioned studies documented the decline in *L. staminea* through discrete sampling periods, only one of these reports quantified changes in *S. gigantea* and *C. nuttallii* populations. Using data from four unique sampling periods between 1974 and 2012 in a coastal Oregon embayment, Strickland et al. (2016) reported a decadal‐scale increase in subtidal *S. gigantea* densities with a slight decline in 2012. Qualitative observations from Prince William Sound, Alaska suggest that no major changes occurred in local *S. gigantea* populations from 2000 to 2007 (Shigenaka et al. 2008). Our results show two predominant patterns in *S. gigantea* population dynamics in the southern Salish Sea. The first is a sequence of gradually increasing biomass and the second is a pattern of biomass increase until ~ 2005 followed by a decline (Fig. 3). Results from the Oregon study appear to mirror our findings regarding *S. gigantea*, quantifying a biomass increase through time (Strickland et al. 2016). Our results, however, also suggest that several populations that experienced extended biomass increases are likely no longer increasing in biomass (i.e., CA, II, PT, WP, and ST; Fig. 3).

*C. nuttallii* populations in Washington inland waters do not appear to be exhibiting any major long‐term synchronous trend in biomass, which is also similar to what has been observed in coastal Oregon populations (Strickland et al. 2016). Whether these trends reflect population patterns elsewhere within the range of these species is unknown due to a lack of additional published literature. Factors affecting recruitment success in another species of cockle, *Cerastoderma edule*, have been shown to be highly site‐dependent (Magalhães et al. 2016). If this were the case for *C. nuttallii*, it may explain the site‐specific patterns in population fluctuations described in this study.

One potential explanatory factor behind the observed population patterns is intra‐ or interspecific competition. While it was beyond the scope of our study to experimentally test competition hypotheses, we suggest that future studies consider indirect interspecific competition, particularly between *L. staminea* and *S. gigantea*, as a tenable explanation for the respective decline and increase in biomass. As *S. gigantea* prefer slightly lower elevations in the intertidal zone and bury deeper than *L. staminea* (Quayle and Bourne 1972; Dethier 2006), perhaps *L. staminea* populations declined for reasons unrelated to *S. gigantea* population change, relaxing indirect competition for food between the species and allowing *S. gigantea* populations to expand. Sea level rise may also promote the extension of *S. gigantea* populations to slightly higher elevations, where they may increasingly overlap with *L. staminea*.

The presence of non‐native clam species, particularly *R. philippinarum*, *Nuttallia obscurata*, and *Mya arenaria*, could also alter population dynamics of native species via direct or indirect competition (Byers 2005; Pranovi et al. 2006; Bendell 2014). *R. philippinarum* populations are enhanced on numerous public beaches within Washington State waters, although they have not naturalized in many of the primary clam habitat areas in the southern Salish Sea. Of the 11 beaches included in this analysis, only one (WP) received significant enhancement of *R. philippinarum* throughout the entire study period. In a separate analysis, we found evidence for negative competitive interactions between *R. philippinarum* and *L. staminea* at this beach (Barber et al. unpubl.), supporting Bendell's (2014) conclusion that enhanced populations of *R. philippinarum* are capable of outcompeting *L. staminea*. Yet, *L. staminea* populations exhibited similar patterns of decline on beaches without *R. philippinarum* in this study (CA and PT) and in Alaska, where the *R. philippinarum* range does not extend (http://invasions.si.edu/nemesis/; accessed 17 July 2018). Thus, interspecific competition with non‐native species may play a much smaller role than factors capable of affecting populations across broad geographic scales. While there is potential that non‐native clams affect individual beach populations of native clam species, it is unlikely that non‐native species are driving the population synchrony found in this study, particularly the range‐wide decline in *L. staminea*.

Although the covariate component of our analysis was exploratory, we were surprised by some of the results. Based on evidence from other bivalve population dynamics research, we hypothesized that a relationship would be found between clam biomass and temperature (e.g., Philippart et al. 2003; Beukema et al. 2009; Magalhães et al. 2016) and/or salinity (e.g., Defeo and de Alava 1995; Baptista and Leitão 2014; Baptista et al. 2014), as both abiotic factors have been found to influence overall clam fitness. Particularly in Puget Sound, we know that decreasing salinity and increasing SST (along with reduced wave energy) leads to a spatial decline in species richness, including bivalve populations (Dethier and Schoch 2005). Because air temperature and freshwater inflows to Puget Sound are correlated with variability in SST and SSS, respectively (Moore et al. 2008), it seems reasonable that these covariates could also play a role in structuring clam biomass (as suggested in Dethier et al. 2012). Moreover, Lowe et al. (2016) found that temporally decreasing salinity in the Salish Sea leads to an increase in the availability of higher‐quality particulate organic matter, an important food source for bivalves. As our results demonstrate, air temperature, Skagit River outflow, and SSS were correlated with clam biomass in the top 15 models (Table 3). However, the best‐fitting model did not include these particular covariates; perhaps the scale of our data was too coarse to detect the effects of more localized variables. Conversely, the combined effects of these environmental variables on clam biomass may be better represented by even larger‐scale processes.

Of the covariates evaluated, the NPGO lagged 4 yr prior to the observation year best explained variability in clam biomass (Table 3). Biomass of all three species was strongly and positively associated with the NPGO (Table 3). Fluctuations in the NPGO, which are largely driven by regional and basin‐scale ocean circulation patterns, explain variations in salinity as well as nitrate and Chl *a* concentrations in coastal areas of the North Pacific Ocean better than other large‐scale climatic patterns (Di Lorenzo et al. 2008). Although the relationship between the NPGO and these environmental parameters is well‐established for coastal areas (Di Lorenzo et al. 2008), it remains unknown how the NPGO affects oceanographic properties within the southern Salish Sea. For the purposes of this study, we have assumed that the relationships described by Di Lorenzo et al. (2008) are similar in our study area, although we recognize that local environmental factors play a strong role in shaping Puget Sound's oceanographic properties (Moore et al. 2008).

This is not the first study to document NPGO effects on invertebrate populations. Decadal change in phytoplankton abundance, as well as mussel and barnacle recruitment, have been linked to the NPGO at multiple sites along the Oregon coast (Menge et al. 2009, 2011). Moreover, Menge et al. (2011) found that phytoplankton abundance was positively correlated with mussel recruitment, indicating bottom‐up forcing of mussel populations. Although a reliable and continuous Chl *a* dataset dating from 1989 to 2016 does not exist for our study area, we know that the NPGO closely tracks Chl *a* and other parameters likely to be important to clam recruitment, growth, and survival in coastal areas. Perhaps the pattern between southern Salish Sea clam populations and the NPGO is driven largely by Chl *a*; where positive NPGO years lead to concomitant changes in Chl *a* (Di Lorenzo et al. 2008), improving the food source for young clams and their subsequent survival.

The lag time associated with the best‐fitting covariate specifies that NPGO effects on clam populations will be evident 4 yr after the settlement process. We estimate that clams targeted in the surveys analyzed here (adults > 38 mm) were generally over 2–3 yr old, depending on the species. Although clam biomass from a single sampling event will be composed of more than one age class of clams, it is logical that the larger clams (i.e., ≥ 3 yr old) in the surveys drive the mean biomass. To verify this, we used median clam length by beach to determine the relative age of clams in our study. *S. gigantea*, *L. staminea*, and *C. nuttallii* median length ranged from 46–92 mm, 33–50 mm, and 29–68 mm, respectively (Table 4). These lengths fall within the range of sizes necessary for *S. gigantea* and *L. staminea* to be approximately 3–5 yr old, although some estimates can vary depending on the tidal elevation and location of the clams used in these length‐age studies (Quayle and Bourne 1972; Houghton 1973). *C. nuttallii* may range in age from 2 to 4 yr old (Quayle and Bourne 1972; Houghton 1973). Despite the variability in predicted age, a 4 yr lag is likely an accurate representation of the general age of these surveyed species. Thus, the approximate age of the surveyed clams supports the possibility that larvae and early settlers are affected by large‐scale environmental variables, including, perhaps, the NPGO.

**Table 4:** Median clam length (mm ± min/max) by beach across all survey years (1989–2016). See Table 1 for beach abbreviations.

	Length (mm)								
	*S. gigantea*			*L. staminea*			*C. nuttallii*		
Beach	Median	Min	Max	Median	Min	Max	Median	Min	Max
CA	66.0	6.9	112.0	42.4	3.5	86.1	68.0	6.4	101.0
WC	68.8	11.8	104.4	41.3	3.6	83.7	49.1	10.5	86.0
SS	75.1	15.0	105.9	40.3	7.0	73.6	54.2	12.7	83.8
SN	69.0	9.0	106.4	35.9	3.7	71.8	55.7	9.8	90.8
II	53.5	7.6	102.6	34.9	4.7	73.5	30.0	9.4	93.8
PT	77.0	8.1	119.6	49.8	5.3	110.1	49.8	9.7	102.8
WP	49.6	10.4	86.3	32.5	7.1	59.7	33.4	11.4	65.2
ST	54.5	5.7	104.4	36.2	4.2	79.4	35.9	8.1	79.2
PS	45.5	5.9	164.5	33.4	4.9	77.3	28.8	5.8	78.3
PD	51.5	6.6	93.1	36.2	5.4	98.7	30.7	10.7	77.4
HI	91.5	9.0	116.9	39.4	7.3	93.2	30.8	14.3	85.9

Many other large or small‐scale drivers not included in this analysis are likely to shape clam population patterns (e.g., bioturbation, disease, ocean acidification [OA], eutrophication, predator populations, etc.). Ocean acidification can alter bivalve neurological functioning, elevate metabolic rates, and reduce shell growth and survivorship (Kroeker et al. 2013; Waldbusser et al. 2015). Although OA is a global issue, upland factors (e.g., land‐use change and freshwater input), sediment characteristics, and water property parameters (e.g., temperature, salinity, depth, alkalinity) can alter the magnitude of OA effects on local carbonate chemistry resulting in highly variable conditions at the local level (Feely et al. 2010). Another possible driver, eutrophication, can effect bivalve populations in numerous ways. For instance, eutrophication can lead to increased ulvoid alga1 blooms, making clams more susceptible to predators as they decrease their burial depth (Auffrey et al. 2004). Perhaps ulvoid mats, which typically are found higher in elevation, negatively affect *L. staminea* or *C. nuttallii* populations and do not impact *S. gigantea* (which prefer lower elevations) as strongly. Finally, large‐scale drivers, such as disease, are capable of devastating a species of clam throughout its entire range. Although *Vibrio tubiashii* has been implicated in significant mortality events of bivalve molluscs (Travers et al. 2015), very little literature exists on how this pathogen may affect these three native clam species; we believe the possibility of disease merits further research.

The trends documented in this study also allow managers to rethink some potential drivers of change that have been proposed as plausible reasons for declines in bivalve populations. For example, decades of shoreline development have eradicated approximately 692 km of Puget Sound's coast (Simenstad et al. 2011). Thus, one could hypothesize that *L. staminea* are more sensitive to shoreline modification than the other two species in our analyses. This hypothesis, however, is difficult to support when two other studies documenting declines in *L. staminea* occurred along relatively undeveloped shorelines in Alaska and British Columbia (Dunham et al. 2007; Shigenaka et al. 2008). Additionally, it seems plausible that heavily harvested beaches would show similar population patterns if overfishing was a problem. Yet the two most heavily harvested beaches in this study, WC and PS, show distinctly different interspecific population patterns by beach. Furthermore, *S. gigantea* and *L. staminea* populations show intraspecific synchrony between a beach closed to fishing (except for incidental tribal harvest, CA) and fished beaches (all other study beaches), indicating that overharvest is probably not driving the population patterns described in this study.

Our finding that spatial and temporal trends in biomass are species‐specific suggests that the species composition of native clams in Washington waters is driven by both large‐scale and local factors. This highlights the complex nature of estuarine systems and the diverse response within a group of organisms to similar environmental conditions. Future investigations could complement our analysis by addressing how local factors impact synchrony in different clam populations. This information could assist managers tasked with the challenge of setting harvest limits on more abundant species while trying to conserve less abundant species undergoing persistent declines. More broadly, however, our study demonstrates the value of long‐term monitoring programs on benthic marine invertebrate populations. These relatively rare programs, combined with data‐driven analyses, should improve scientists’ ability to determine the difference between episodic change quantified within shorter temporal scales and more persistent ecological change across multidecadal scales. Such information will be vital in tracking the biological impacts of global changes at the local level and can promote the sustainable management of invertebrate populations under future ocean conditions.

## Conflict of Interest

None declared.

## Supporting information


**Table S1**. DFA models comparing data support for models fit with one to three trends (M) and two different forms of the factor loadings matrix (Z). In the unconstrained form of Z, each population is assigned a unique factor loading on each of the modeled trends, whereas in the constrained form, geographically proximate populations are assigned equal loadings on a given trend.
**Fig. S1**. Model innovations for the best‐fitting DFA model with three trends and no covariates explaining temporal variability in clam biomass. See Table 1 for a key to beach name abbreviations.
**Fig. S2**. Autocorrelations versus temporal lags for innovations from the best‐fitting DFA model with three trends and no covariates explaining temporal variability in clam biomass. Horizontal dashed blue lines indicate the upper and lower 95% confidence intervals. See Table 1 for a key to beach name abbreviations.
**Fig. S3**. Quantile‐quantile plots of innovations from the best‐fitting DFA model with three trends and no covariates explaining temporal variability in clam biomass. See Table 1 for a key to beach name abbreviations.
**Fig. S4**. Pairwise Spearman's rank correlations among time series of model innovations generated from the best‐fitting DFA model (Model 1, Table 3). The color of each box shows the strength of the correlation between a population in row *i* and a population in column *j* of the matrix. See Table 1 for a key to beach name abbreviations.
