# Supplementary material for: Intertidal clams exhibit population synchrony across spatial and temporal scales
Source: Limnol Oceanogr. 2018 Dec 3;64(Suppl 1):S284–300. doi: 10.1002/lno.11085 (PMC6472620; doi:10.1002/lno.11085)
Supplement: Supplementary file 1 — Table S1. DFA models comparing data support for models fit with one to three trends (M) and two different forms of the factor loadings matrix (Z). In the unconstrained form of Z, each population is assigned a unique factor loading on each of the modeled trends, whereas in the constrained form, geographically proximate populations are assigned equal loadings on a given trend. Fig. S1. Model innovations for the best‐fitting DFA model with three trends and no covariates explaining temporal variability in clam biomass. See Table 1 for a key to beach name abbreviations. Fig. S2. Autocorrelations versus temporal lags for innovations from the best‐fitting DFA model with three trends and no covariates explaining temporal variability in clam biomass. Horizontal dashed blue lines indicate the upper and lower 95% confidence intervals. See Table 1 for a key to beach name abbreviations. Fig. S3. Quantile‐quantile plots of innovations from the best‐fitting DFA model with three trends and no covariates explaining temporal variability in clam biomass. See Table 1 for a key to beach name abbreviations. Fig. S4. Pairwise Spearman's rank correlations among time series of model innovations generated from the best‐fitting DFA model (Model 1, Table 3). The color of each box shows the strength of the correlation between a population in row i and a population in column j of the matrix. See Table 1 for a key to beach name abbreviations. [file LNO-64-S284-s001.docx]

**Table S1**. DFA models comparing data support for models fit with one to three trends (M) and two different forms of the factor loadings matrix (Z). In the unconstrained form of Z, each population is assigned a unique factor loading on each of the modeled trends, whereas in the constrained form, geographically proximate populations are assigned equal loadings on a given trend.

| M | Z | k | delta AICc | AICc weight |
| --- | --- | --- | --- | --- |
| 2 trends | constrained | 44 | 0.00 | 8.58E-01 |
| 3 trends | constrained | 63 | 3.64 | 1.39E-01 |
| 3 trends | unconstrained | 99 | 12.01 | 2.12E-03 |
| 2 trends | unconstrained | 68 | 14.04 | 7.66E-04 |
| 1 trend | constrained | 24 | 80.51 | 2.82E-18 |
| 1 trend | unconstrained | 36 | 92.51 | 7.01E-21 |

**
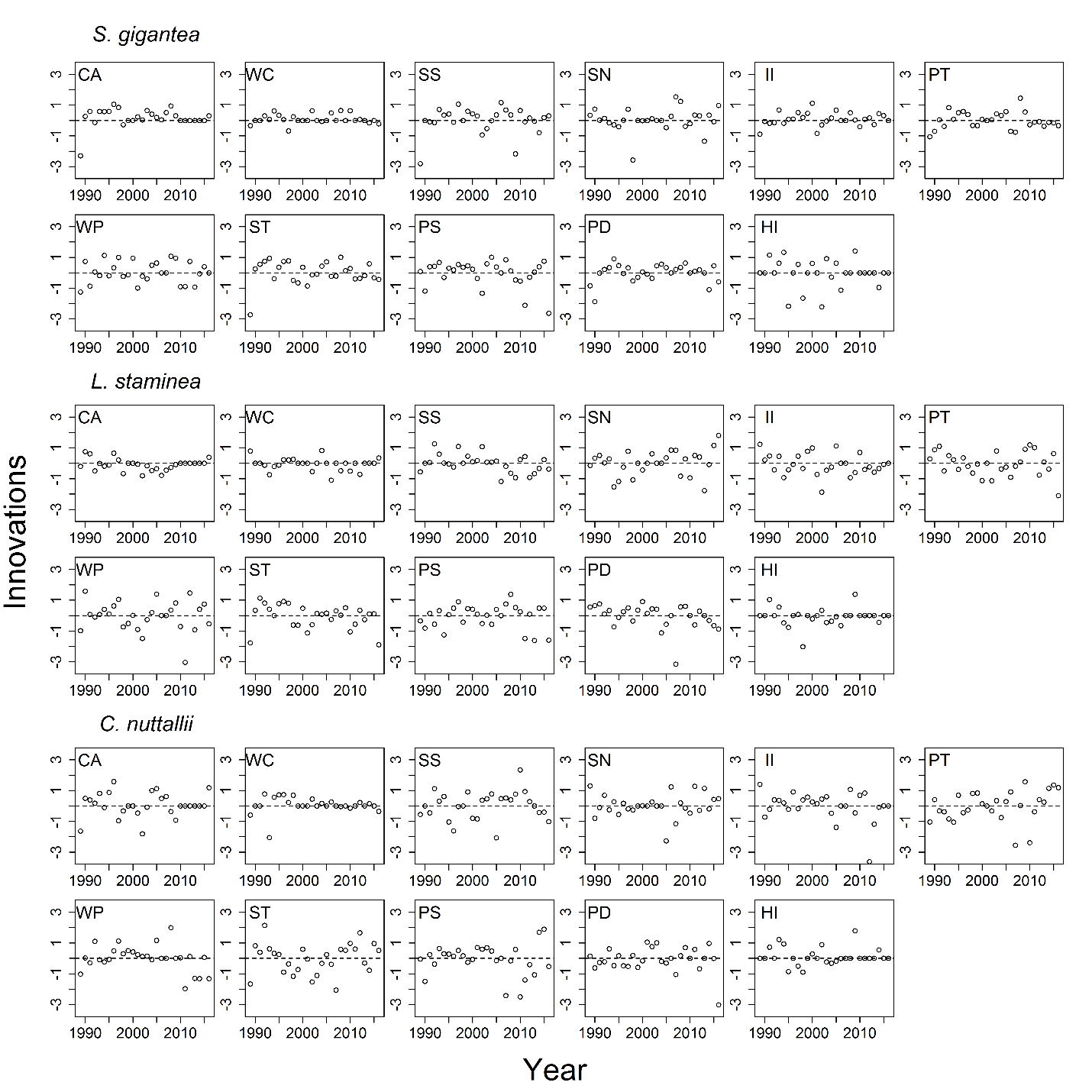
**

**Figure S1**. Model innovations for the best-fitting DFA model with three trends and no covariates explaining temporal variability in clam biomass. See Table 1 for a key to beach name abbreviations.

**
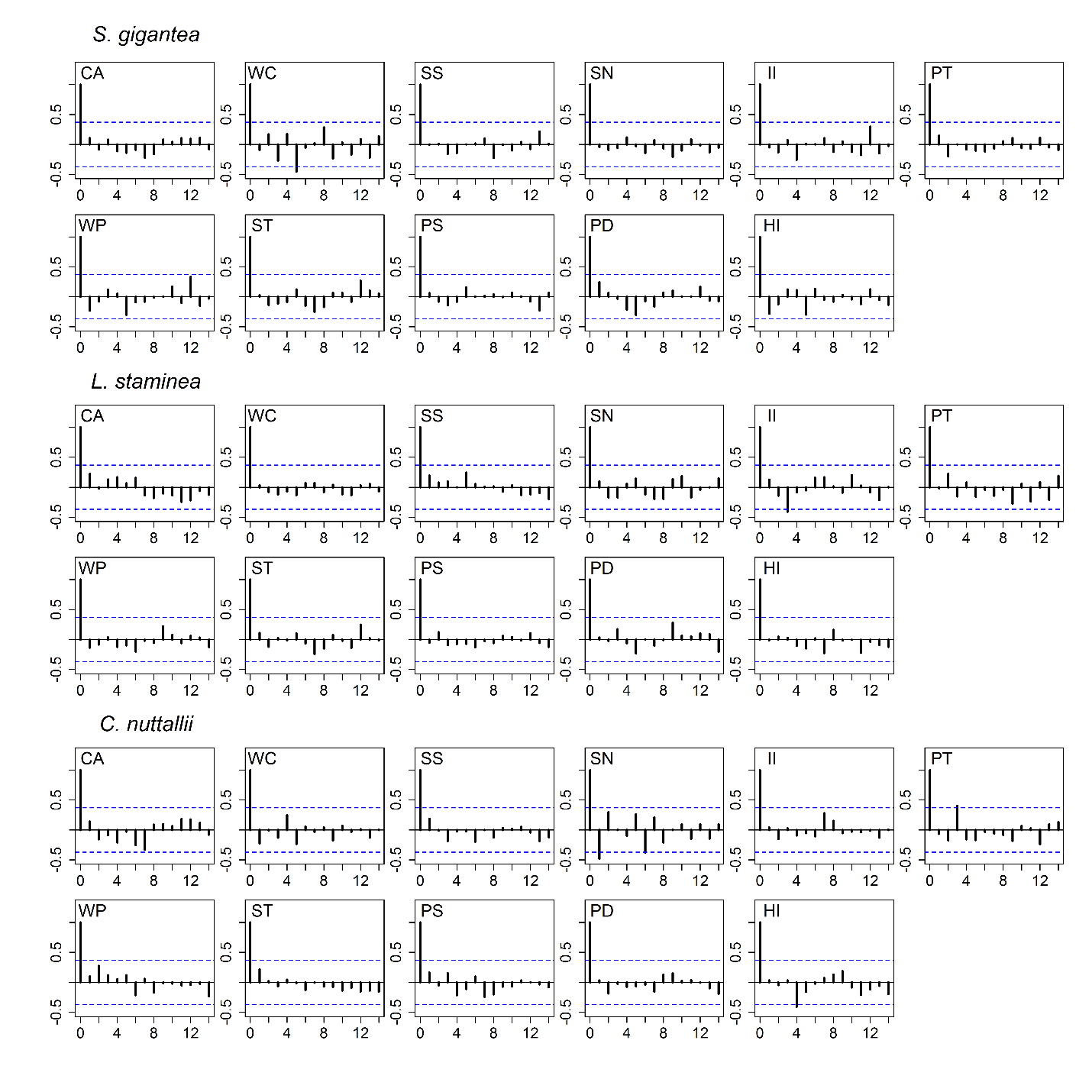
**

**Figure S2**. Autocorrelations versus temporal lags for innovations from the best-fitting DFA model with three trends and no covariates explaining temporal variability in clam biomass. Horizontal dashed blue lines indicate the upper and lower 95% confidence intervals. See Table 1 for a key to beach name abbreviations.

**
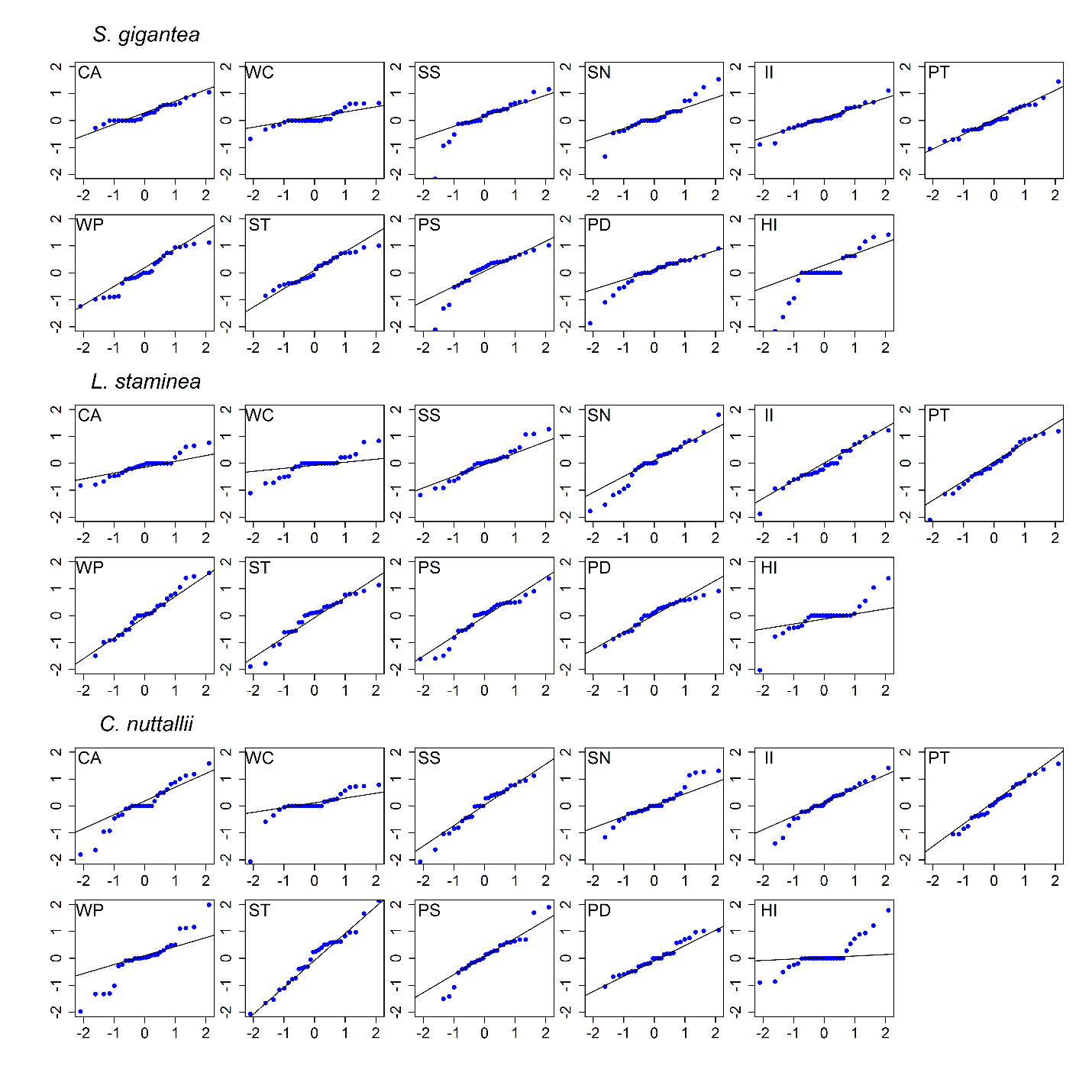
Figure S3**. Quantile-quantile plots of innovations from the best-fitting DFA model with three trends and no covariates explaining temporal variability in clam biomass. See Table 1 for a key to beach name abbreviations.

**
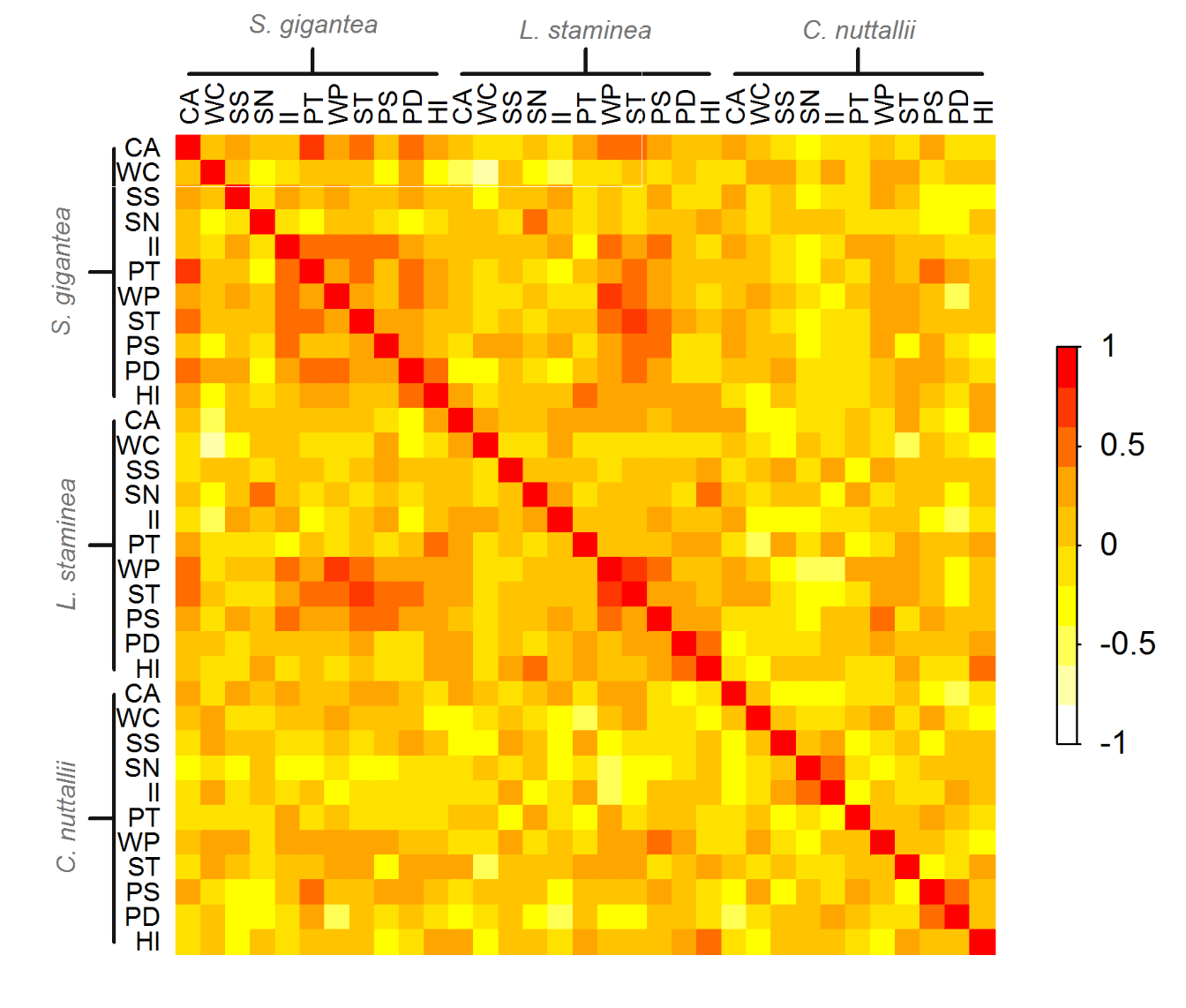
**

**Figure S4**. Pairwise Spearman’s rank correlations among time series of model innovations generated from the best-fitting DFA model (Model 1, Table 3). The color of each box shows the strength of the correlation between a population in row *i* and a population in column *j* of the matrix. See Table 1 for a key to beach name abbreviations.
